# Prevalence of Problem Gambling Among Women Using Shelter and Drop-in Services

**DOI:** 10.1007/s11469-021-00524-z

**Published:** 2021-03-15

**Authors:** Flora I. Matheson, Parisa Dastoori, Tara Hahmann, Julia Woodhall-Melnik, Sara J. T. Guilcher, Sarah Hamilton-Wright

**Affiliations:** 1 MAP Centre for Urban Health Solutions, St. Michael’s Hospital, 30 Bond Street, Toronto, Ontario M5B1W8 Canada; 2 Dalla Lana School of Public Health, Centre for Criminology and Socio-legal Studies, University of Toronto, Toronto, Ontario Canada; 3 Department of Social Science, University of New Brunswick, 100 Tucker Park Rd., PO Box 5050, Saint John, E2L 4L5 Canada; 4 The Leslie Dan Faculty of Pharmacy, University of Toronto, 144 College St, Toronto, Ontario M5S 3M2 Canada

**Keywords:** Gambling, Poverty, Homelessness, Shelter and drop-in services, Women, Prevalence

## Abstract

People experiencing poverty/homelessness have higher rates of problematic gambling than the general population. Yet, research on gambling among this population is sparse, notably among women. This study examined prevalence of problematic gambling among women using shelter and drop-in services in Ontario, Canada. The NORC Diagnostic Screen for Disorders was administered to women during visits to 15 sites using time/location methodology. Within a sample of 162 women, the prevalence of at-risk (6.2%), problem (9.3%), and pathological gambling (19.1%) was higher than the general population. Among women who scored at-risk or higher, 55.4% met criteria for pathological gambling. The findings suggest that women seeking shelter and drop-in services are vulnerable to problematic gambling. Creating awareness of this vulnerability within the shelter and drop-in service sector is an important first step to support women with gambling problems who face financial and housing precarity.

## Introduction

Although historically a male-dominated activity, women are increasingly more involved in gambling (McCarthy et al., 2018; McKay, 2005; van der Maas et al., 2018). While rates of problem gambling are higher among men than women across jurisdictions (Williams R. J. et al., 2012), growth in participation among women is attributed to aggressive advertising campaigns that are increasingly directed toward them (Kairouz et al., 2017) and to an increase in the variety of available gambling options (McCarthy et al., 2018; Volberg, 2003; Wardle, 2017). Online gambling participation is increasing more rapidly among women than men and the harms that result are greater than previously expected (Edgren et al., 2017; McCormack et al., 2014).

Emerging research suggests there is a relationship between problem gambling and homelessness (Lepage et al., 2000; Matheson et al., 2014; Nower et al., 2015; Sharman et al., 2015). The prevalence of problem gambling among those experiencing homelessness (primarily male samples) is up to 9-fold higher than the general population (Lepage et al., 2000; Matheson et al., 2014; Nower et al., 2015; Sharman et al., 2015). With a primary focus on male gambling in the literature, there remains a critical gap in knowledge on prevalence of gambling problems among women experiencing poverty and homelessness.

The preponderance of research on men within the field of problem gambling, coupled with little research with people experiencing marginalized statuses, calls for more concentrated efforts to understand women's experiences, especially in relation to poverty and homelessness. Families and women represent the fastest growing sectors of the homeless population in the USA (Welch-Lazoritz et al., 2015). In Canada, women represent 27.3% of the homeless population (Gaetz et al., 2016). Women are at greater risk for economic disadvantage making them vulnerable to homelessness (Baker et al., 2010; O’Campo et al., 2015; Pavao et al., 2015; Woodhall-Melnik et al., 2016), while the constraints of poverty can potentially intensify existing gambling tendencies (Sharman et al, 2016). These forms of precarity affect women’s psycho-social well-being producing feelings of isolation, stress, and other mental health concerns, which in addition to low socioeconomic status are factors associated with problem gambling (Afifi et al., 2010; Hing, Russell, Tolchard, et al., 2016; Järvinen-Tassopoulos, 2016; Morrison, 2017). Researchers note that women progress to gambling-related problems more quickly than men and at later stages of life (Grant et al., 2012; Merkouris et al., 2016). Research indicates that women who have gambling concerns progress to financial crisis earlier than do men; in general, they earn less and are less likely to have access to credit and to financial reserves than men (Kairouz et al., 2017; Lesieur & Blume, 1991; Potenza et al., 2001). Economic instability also means that women have fewer financial reserves to sustain gambling losses thereby potentially shouldering a greater burden of gambling harms. It is possible that rates of problem gambling among women experiencing financial and housing precarity mirror the higher rates suggested in studies among their male counterparts, but to date no studies have explored this possibility. The objective of this paper is to describe findings from a rapid screening study of problem and pathological gambling among women frequenting shelters and drop-ins in two large Canadian cities.

## Materials and Methods

### The Setting

The study was conducted in two large urban centers in Ontario, Canada, at emergency shelters and drop-in programs for women and families. The research team engaged the community service sector through two umbrella and 8 individual operators of shelters and drop-in programs. These operators provided expertise on the shelter environments and recommendations on approaches to access the facilities for the purpose of this research. In Hamilton, the research team engaged 4 shelters and 2 drop-in programs. In Toronto, the team engaged 7 shelters and 2 drop-in programs. Individual operators were identified through an online list of shelter and housing services available across the cities of Hamilton and Toronto.

The two umbrella operators were Good Shepherd Centre in Hamilton and the City of Toronto. Good Shepherd Centre is the largest provider of human and social services for people who live in the Greater Hamilton area. They provide access to meal programs, emergency shelters, and supportive housing for a variety of populations that experience housing instability and poverty. The City of Toronto funds and operates housing and homelessness services in Toronto including emergency and transitional shelters, respite centers, and manages the wait lists for affordable housing. These shelters and drop-in programs see a range of women across all ages for assistance with family homelessness, pregnancy, violence, addictions (including harm reduction), meals and recreational activities, healthcare, housing support, clothing and household items, and crisis services. Women have varied engagement with services and may access services at multiple sites in both urban areas.

### The NORC Diagnostic Screen for Disorders

Lifetime gambling problems were assessed using The NORC Diagnostic Screen for Disorders (NODS) (Hodgins, 2004; Toce-Gerstein et al., 2009; Wickwire Jr et al., 2008). The NODS is a reliable and valid instrument with high internal consistency as well as good concurrent and discriminant validity (Hodgins, 2004; Wickwire Jr et al., 2008) with a hierarchically structured 17-item screening process that yields a score ranging from 0 to 10 (Hodgins, 2004; Toce-Gerstein et al., 2009; Wickwire Jr et al., 2008). The screen assesses ten criteria for disordered gambling that are derived from the Diagnostic and Statistical Manual of Mental Disorders (DSM-V) including preoccupation, tolerance, withdrawal, loss of control, escape, chasing, lying, illegal acts, risked relationships, and need for bailout. The NODS tool includes three questions (the “NODS-CLiP”) to screen out non-problem gambling early on in the interview process. Those with a score of 1 or more on the CLiP are administered the remaining questions on the NODS. Table 1 displays the relationship between NODS scores and gambling status. A null score on the NODS is reflective of non-problematic levels of gambling. A score of 1 or 2 indicates mild subclinical risk for gambling problems (at-risk gambling), 3 or 4 suggests moderate subclinical risk (problem gambling), while 5+ suggests likely diagnosis of pathological gambling problems (pathological gambling).

**Table 1:** NODS categories generated by the scoring algorithm

Score	Description	Subgroup name
0	Non-problematic levels of gambling	Non-problem gambling
1 or 2	Mild subclinical risk for gambling problems	At-risk gambling
3 or 4	Moderate subclinical risk for gambling problems	Problem gambling
5 +	Likely diagnosis of pathological gambling	Pathological gambling

### Participant Sampling and Recruitment

We used an adaptation of the time/location sampling strategy(Baggett, Campbell, et al., 2016; Baggett, Rigotti, et al., 2016; Karon, 2005; Matheson et al., 2014; Shaghaghi et al., 2011) to ensure we recruited women accessing a variety of services and programs. This sampling methodology, which involves multiple visits to recruit at each location on different days and times, proved effective in recruiting men experiencing homelessness for a problem gambling prevalence study in Canada (Matheson et al., 2014). Recruitment of women occurred between July 9, 2018, and March 17, 2019. Research staff visited 15 shelters and drop-in programs to recruit the study participants. The total number of visits to shelters was 39. The recruiters approached people at two time periods, from 10 am to 3 pm (77%) and from 4 pm to 8 pm (33%). Scheduling recruitment visits on different days and at different times ensured that the sample would include women who accessed the sites on different days of the week (weekdays and weekends) and at various times of day (morning and afternoon). Staff posted information flyers prior to the first onsite visit by the research team. The recruiters asked all women they encountered to participate in the study.

### Data Collection

Each participant was asked to choose an onsite location (e.g., office or common area) to answer the NODs questions. Potential participants received a study information and consent sheet and provided verbal consent to participate, which was recorded on an encrypted tablet. In an effort to conduct rapid onsite screening for problem gambling among women using shelter and drop-in services, we did not collect information (e.g., socio-demographic) other than what was included in the screener. This was a similar process to another study, which examined prevalence of problem gambling among men (Matheson et al., 2014). The team used SurveyMonkey® for tablet-based data collection. After obtaining verbal consent, recruiters read out the following definition of gambling and then asked participants to indicate if they had ever gambled based on the definition:

**Gambling** is when someone makes a bet on an event with an uncertain outcome in the hopes of winning money or valuables. There are a variety of activities that may be considered gambling such as, playing cards (i.e. blackjack) or the slot machines at a casino, internet gambling, and placing bets on horses/dogs at a race track, or through places with off-track betting. Also, there are less obvious forms of gambling such as playing the lottery, scratch cards or pull-tabs, betting money at a bingo hall, placing money in the stock market, and playing cards with friends.

Participants who indicated they never gambled were thanked for their time and the screening ended. Those who indicated that they had participated in gambling were then asked the questions on the CLiP. Women who answered yes to at least one of the CLiP questions completed the full NODS.

### Data Analysis

Data were downloaded from SurveyMonkey. SAS 9.4 software was used to conduct data analysis. Based on a pre-defined algorithm, the NODS items were summed across participants. The scores were grouped into the categories described in Table 1. Counts and proportions were calculated for each of the categories. This study was approved by the Research Ethics Board of St. Michael’s Hospital, Toronto, Ontario.

## Results

The recruiters approached 386 women and 217 refused to participate (*n* = 169, 56.2%). Seven participants who initially indicated interest in participating did not complete the three NODS-CliP questions; therefore, their data were removed from the study. The final sample included 162 women.

Table 2 reports data on lifetime gambling activities of 162 participants based on NODS scores. Among the 162, 30.3% of the sample identified as non-gambling and 35.2% as non-problem gambling. Those categorized as at-risk, problem, and pathological gambling represented 34.6% of the sample (6.2%, 9.3%, and 19.1%, respectively). Of the women (*n* = 56) with gambling problems, 17.9% scored in the category of at-risk gambling, 26.8% as problem gambling, and 55.4% as pathological gambling.

**Table 2:** Lifetime gambling status based on the NODS (*n* = 162)

	Non-gambling % (*n*)	Non-problem gambling % (*n*)	At-risk gambling % (*n*)	Problem gambling % (*n*)	Pathological gambling % (*n*)
Full sample	30.3 (49)	35.2 (57)	6.2 (10)	9.3 (15)	19.1 (31)
Sub-sample with gambling problems			17.9 (10)	26.8 (15)	55.4 (31)

## Discussion

This study examined lifetime prevalence of at-risk, problem, and pathological gambling among women using shelter and drop-in services in Toronto and Hamilton, Ontario. Among the entire sample, 28.4% met the criteria for lifetime problem and pathological gambling and 6.2% met the criteria for at-risk gambling. Among women who scored at-risk or higher, 82.2% experienced lifetime problem or pathological gambling. This is the first study we know of that examined prevalence of problem gambling among women in these settings. The findings underscore a serious and hidden public health issue, namely the confluence of problem gambling and poverty/homelessness among women. Earlier evidence suggested that prevalence of problem gambling is higher among those experiencing homelessness (in mostly male samples) with reported lifetime rates of subclinical and disordered gambling as high as 58% (Lepage et al., 2000; Matheson et al., 2014; Nower et al., 2015; Shaffer et al., 2002; Sharman et al., 2015) contrasted with general population prevalence rates that range between 0.5 and 7.6% (Stucki & Rihs-Middel, 2007; Williams R. J. et al., 2012).

Studies examining gender differences among the general population (Carneiro et al., 2019; Wong et al., 2013) and among those experiencing homelessness (Shaffer et al., 2002) report higher problem gambling rates among men than women. When comparing current study findings with that of a similar study conducted in Ontario, Canada (Matheson et al., 2014), the prevalence of lifetime problem and pathological gambling, among a predominately male sample experiencing homelessness, was slightly higher (34.1%) than our finding of 28.4% among this sample of women. Rates of at-risk gambling were similar among men and women as per the 2014 and current study (8.3% and 6.2%, respectively).

In light of the precarious social and financial situations that many women experience, somet may seek the financial freedom that gambling promises, yet they may also bear a greater burden of the economic costs that arise from problematic gambling (Järvinen-Tassopoulos, 2016; Morrison, 2017; Potenza et al., 2006; Rash & Petry, 2017). Women may turn to gambling as physical and emotional refuge to escape life stressors and painful memories (Nixon et al., 2013). Gambling may also help women cope with social isolation, loneliness, physical health problems (McCormack et al., 2014; Pattinson J & A., 2017), and anxious and depressive symptomology (Jauregui et al., 2017).

Screening, along with tailored treatment for problem gambling, is essential to ensure women using shelter and drop-in services have access to appropriate care to address gambling in conjunction with financial vulnerability, homelessness, and poor health (e.g., psychiatric, substance use, and posttraumatic stress disorders) (Billi et al., 2014; Langan et al., 2019; Nower et al., 2015; Shaffer et al., 2002). Qualitative studies suggest that adult gambling and homelessness occur in combination with mental illness, substance use, interpersonal problems, unemployment, domestic violence, criminal involvement, and discrimination, and can be pre-dated by childhood abuse, neglect, and homelessness (Hamilton-Wright et al., 2016; Holdsworth & Tiyce, 2013). When gambling and housing problems fuse, already poor mental health and substance use are worsened, while the weight of these complex conditions often thwarts efforts to secure and maintain safe housing.

### Strengths and Limitations

A major strength of this study is that, to our knowledge, this is the first study to examine prevalence of gambling problems specifically among women seeking shelter and drop-in services. The study was conducted within the shelter and drop-in services sectors in two large urban centers using a modified time/location methodology. A limitation of the study that has implications for generalizability is that it is comprised of women in two Ontario cities who volunteered to answer questions about gambling. Random sampling was not possible as there was no official register for shelter or drop-in centers in Ontario, Canada. The findings may not be representative of women who experience homelessness and poverty in other areas of Ontario, other provinces or countries, or in suburban and rural areas. The findings represent women volunteering to participate from two large urban centers in Ontario that are marked by a lack of safe and affordable housing and considerable homelessness. The findings suggest a need for additional studies that capture prevalence rates in diverse populations of women (e.g., racially/ethnically diverse, sex/gender diverse) and across multiple geographic contexts.

Women who experience gambling problems are highly attuned to associated stigma related to their gambling activity and this can affect their willingness to report gambling concerns and seek treatment (Baxter et al., 2016; Hahmann et al., 2020). Given we did not have an opportunity to visit programs to build rapport across the sites, we are grateful for those who chose to participate. We also could not provide honorariums for the women who participated in the prevalence study, which might have increased participation. It is important to note that the rate of refusals may illuminate the underlying concerns women have in revealing their gambling activities (e.g., fear of stigma) as suggested by other researchers (Hing, Holdsworth, et al., 2014; Holdsworth & Tiyce, 2012). This would be important to explore in future research. Studies are also needed to confirm that the findings are reproducible in other settings among women experiencing poverty and homelessness. The findings mirror those of similar studies with primarily male samples indicating that gambling may be of particular concern among people experiencing poverty/homelessness (Hing, Russell, Tolchard, et al., 2016; Lepage et al., 2000; Matheson et al., 2014; Shaffer et al., 2002; Sharman et al., 2015). One limitation is that we are unsure whether women we approached and who refused to participate did so because they did not want to talk about their gambling (e.g., fear of disclosure) (Baxter et al., 2016; Hing, Russell, & Gainsbury, 2016; Hing, Russell, Gainsbury, et al., 2016) or because of issues of trust. Trust may have affected the participation rate. We approached women in emergency shelters and drop-in centers, environments that provide services for mobile populations. The women seeking such services will have had histories of trauma, mental illness and addictions, and be untrusting of service providers and researchers (Oudshoorn, Ward-Griffin, Forchuk, et al., 2013; Oudshoorn, Ward-Griffin, Poland, et al., 2013; Sundin & Baguley, 2015).

### New Directions

Our current understanding of gambling and poverty/homelessness indicates that services need to be client-centered offering wrap-around services that are gender-, cultural-, and trauma-specific (Guilcher et al., 2016; Guilcher et al., 2020; Hamilton Wright et al., 2019; Woodhall-Melnik et al., 2019) while addressing the complex underlying health issues women face (Holdsworth et al., 2012; Holdsworth et al., 2013; Pattinson J & A., 2017). To this end, the Jean Tweed Centre in Ontario provides services to women experiencing gambling problems with a focus on multiple health and social needs using trauma-informed and trauma-specific approaches to care. Good Shepherd Ministries, also in Ontario, designed and initiated the first problem gambling addiction program situated in a shelter service in Canada to address the co-concerns of problem gambling and homelessness/poverty among adult men and women. This program is unique, as it is offered in a shelter service setting and it is part of a suite of services available to clients to address homelessness, addiction, financial instability, and mental health concerns, following a holistic approach to care.

Women differ in their pathways into gambling, how they seek treatment, and respond to care (Holdsworth et al., 2012; Holdsworth & Tiyce, 2013; McMillen et al., 2007), important considerations when developing services for women who experience poverty and homelessness. Screening for problem gambling among this sub-sector of women can be challenging as there is considerable stigma associated with gambling (Horch & Hodgins, 2015). Yet, screening is imperative to generate awareness of problem gambling in social services settings, which can then be mobilized to develop and implement interventions specific to women and to advocate for gambling-treatment specific funding for women.

A gender-responsive framework for care will acknowledge how women’s pathways into gambling and their help-seeking differ from men and within diverse groups of women. Several studies from across disciplines (gambling, addiction, criminal justice) point to the need for gender-responsive treatment that addresses the unique needs and experiences of women (Boughton, 2003; Boughton et al., 2017; Covington & Bloom, 2007; Covington et al., 2008; Wright et al., 2007). Future research must gather knowledge on the needs of women who seek shelter services to better tailor treatment to their unique situations. We lack sufficient knowledge on the nature of the intersection between gambling, poverty, and homelessness among women. Future research should expand its focus to include gender and sexual diversity and people experiencing structural marginalizationd to get a fuller understanding of gambling prevalence (e.g., large-scale studies to capture diverse groups and peoples). While prevalence data provides necessary perspective on the extent of the problem, thereby indicating a need for screening and treatment (in-house or referral) for gambling problems within shelter and drop-in agencies that serve women, equally important is a deeper understanding of where gambling fits in the larger context of adverse life histories.

## Conclusions

The prevalence of problem and pathological gambling is high among women seeking shelter and drop-in services. The path into problem gambling is most likely complex and multidimensional—connected to early childhood and adult experiences of trauma, psychiatric, and substance use disorders. Lack of affordable and safe housing complicates problem gambling specific treatment for women. Cross-sectoral screening and treatment is needed to enhance integration and coordination of services for those experiencing homelessness (e.g., shelter and addiction services, criminal justice and mental health services, housing, family, and immigration services) and to address the unique experiences of women (Holdsworth et al., 2012; Holdsworth et al., 2013; Pattinson J & A., 2017).

## Data Availability

Under research ethics guidelines we are not able to share confidential study data.
